# Dietary supplementation with *Acremonium terricola* culture alters the gut microbial structure and improves the growth performance, antioxidant status, and immune function of weaning piglets

**DOI:** 10.1186/s12917-023-03778-y

**Published:** 2023-12-06

**Authors:** Wei Wang, Yizhu Peng, Yong Nie, Yulong Wang, Chuang Wang, Bo Huang

**Affiliations:** 1 Anhui Provincial Key Laboratory of Microbial Pest Control, Anhui Agricultural University, Hefei, 230036 China; 2 Jiangxi Zhengbang Academy of Agricultural Sciences, Nanchang, 330000 China; 3 School of Civil Engineering and Architecture, Anhui University of Technology, Ma’anshan, 243002 China

**Keywords:** *Acremonium terricola* culture, Weaning piglets, Growth performance, Immunity, Gut microflora

## Abstract

**Background:**

*Acremonium terricola* is used in the feed of dairy animals to promote growth and control diseases. However, the effects of dietary supplementation with *A. terricola* on the gut microbial structure of weaning piglets remain poorly understood. Therefore, in this study, we investigated the effects of dietary supplementation with *A. terricola* culture (ATC) on the growth performance, antioxidant status, immunity, and gut environment of weaning piglets. Sixty piglets were fed a basal diet supplemented with 1 g ATC/kg of basal diet (experimental group). Another 60 piglets did not receive ATC (control group). The intervention lasted for 20 days.

**Results:**

The experimental group had higher daily weight gain and feed efficiency than did the control group. Significant increases were noted in the levels of serum insulin (*P* = 0.0018), insulin-like growth factor (*P* = 0.0018), triiodothyronine (*P* = 0.0031), immunoglobulin A (*P* < 0.0001), immunoglobulin M (*P* = 0.001), immunoglobulin G (*P* = 0.0001), and interferon γ (*P* < 0.0001) in the experimental group compared with the levels in the control group. Furthermore, ATC supplementation significantly reduced (*P* < 0.05) the relative abundance of *Shuttleworthia*, *Succinivibrio*, *Roseburia*, *Ruminococcus*, and *Paludibacter* but increased that of *Phascolarctobacterium*, *Megasphaera*, *Faecalibacterium*, and *Prevotella* in the experimental group compared with that in the control group. Notably, ATC supplementation significantly increased the relative abundance of *Faecalibacterium prausnitzii* (*P* < 0.05), which is involved in anti-inflammatory activities, gut barrier enhancement, and butyrate production.

**Conclusions:**

Dietary supplementation with ATC may improve the growth performance, antioxidant status, immunity, and fecal microflora of weaning pigs.

## Background

In pig farming, postweaning is a crucial and challenging life stage because pigs are highly susceptible to nutritional, social, and environmental stress and gastrointestinal disorders, which result in growth retardation and even death [1]. Over the last decades, promoters of nutrient digestion and absorption have been widely used in pig farming to improve the growth performance, health, and well-being of weaning pigs; however, the use of such promoters has led to the development of antibiotic resistance against some pathogens [2]. An inextricable relationship has been observed between pigs, particularly weaning pigs, and their gut microbiota, which is involved in nutrient digestion and absorption, gastrointestinal development, energy metabolism, and immunologic functions [3, 4]. Gut microbiota can adapt to changes in dietary factors, thus helping the host to better adapt to different feeds [3].

The use of feed additives, such as lactic acid bacteria, *Bacillus*, *Cordyceps*, and yeast, is an effective strategy for the prevention of various diseases and the improvement of weight gain and immunity in animals [5, 6]. Dietary supplementation with *Lactobacillus acidophilus* significantly increased the growth rate of piglets without affecting their feed intake [7]. A molecular investigation revealed that *L. amylovorus* suppressed the Toll-like receptor (TLR)4 signaling pathway in the jejunum of piglets, and that *L. casei* downregulated enterotoxigenic *Escherichia coli* (ETEC)–induced expression of TLR4, TLR2, interleukin (IL)-17, and tumor necrosis factor-α in jejunal tissues [8, 9]. In piglets, live yeast supplementation reduced ETEC K88–induced inflammation by downregulating the expression of IL-1β and nuclear factor-κB [10]. Weaned pigs fed *Cordyceps militaris* exhibited improved growth performance; enhanced immunoglobulin secretion; and reduced inflammation, pathogenic population, and cholesterol concentrations [11, 12].

*Acremonium terricola* (Ascomycota) is an important pathogenic fungus [13]. Similar to *C. gunnii* (the rare Chinese caterpillar fungus), *A. terricola* has bioactive compounds, including cordycepin, cordycepic acid, D-mannose, D-galactose, aspartic acid, and glutamic acid [14]. *A. terricola* culture (ATC) has diverse biological functions, such as antioxidant, immune regulatory, and anti-inflammatory properties [15]. ATC supplementation may improve production, antioxidant status, energy balance, and apparent nutrient digestibility and may increase the relative population size of proteolytic bacteria and protozoa in cows [14, 15]. ATC supplementation of basal diets enhanced the growth performance and antioxidant properties of Hortobagy geese [16]. It further improved average daily gain (ADG), biochemical parameters, antioxidant properties, and immunity in the serum and liver of Sprague Dawley rats [17]. Therefore, in piglets, ATC supplementation may improve growth performance and immune function.

Although ATC accelerates weight gain and immunity in piglets, data regarding the gut microbiota and blood parameters of piglets fed ATC remain limited [18]. We hypothesized that ATC supplementation improves the growth performance, antioxidant status, immunity, and gut health of weaning piglets. Therefore, in the present study, we investigated the effects of dietary supplementation with ATC on the gut microbiota, growth performance, antioxidant status, and blood biochemistry of weaning piglets.

## Results

### Growth performance

Compared with the piglets who did not receive ATC (control group), those supplemented with ATC (experimental group) exhibited significant increases in ADG during the experimental period (*P* = 0.0106). However, no significant change was noted in average daily feed intake (ADFI; Table 1). Furthermore, the feed conversion ratio (FCR, calculated as ADG divided by ADFI) was lower in the experimental group than in the control group (*P* = 0.001).

**Table 1:** Effects of Acremonium terricola culture supplement on growth performance of weaned pigs

Item	Control groups			Treatment groups			SEM	*p* value
Number of pigs	20	20	20	20	20	20		
Body weight (kg)	11.75	11.8	11.8	11.8	11.75	11.8	0.02887	0.933
0–10 days								
ADFI ^a^ (g/pig/day)	637.7	609.2	605.0	618.3	612.3	571.1	10.76	0.406
ADG ^b^ (g/pig/day)	368.4	352.5	370.2	467.1	456.4	454.8	5.766	0.004
FCR^c^ (kg feed/kg gain)	1.7	1.7	1.6	1.3	1.3	1.3	0.03333	0.008
11–20 days								
ADFI (g/pig/day)	819.2	844.1	793.4	866.3	850.6	851.0	15.58	0.14
ADG (g/pig/day)	422.2	401.3	410.2	506.3	473.8	487.3	3.372	0.002
FCR (kg feed/kg gain)	1.9	2.1	1.9	1.7	1.8	1.7	0.03333	0.02
Overall								
ADFI (g/pig/day)	728.5	726.6	699.2	742.3	731.5	711.0	2.696	0.064
ADG (g/pig/day)	417.8	404.4	412.5	464.2	437.5	448.8	4.008	0.011
FCR (kg feed/kg gain)	1.7	1.8	1.7	1.6	1.7	1.6	0.01	0.001

a ADFI: Average daily feed intake

b ADG: Average daily gain

c FCR: Feed conversion ratio

### Blood profiles

**Fig. 1:**
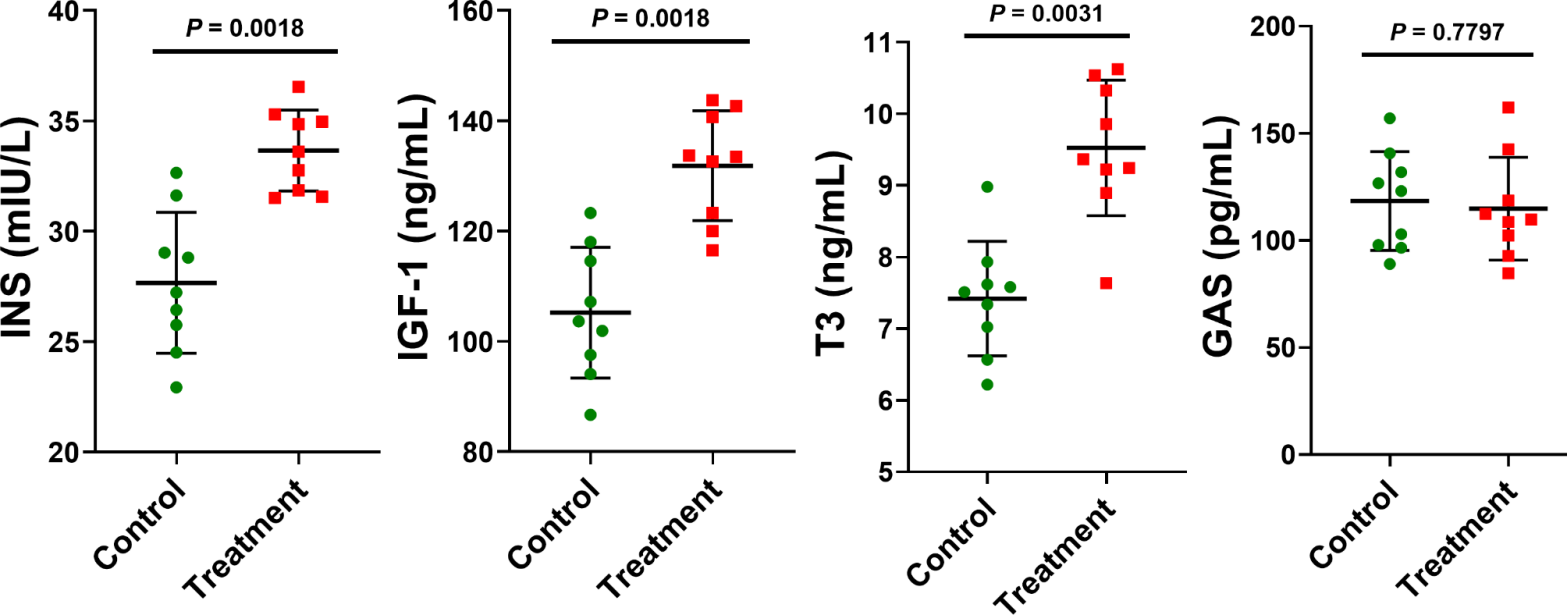
Effects of dietary supplementation with ***Acremonium terricola*culture on factors influencing the feed intake of weaning piglets.** INS, serum insulin; IGF, insulin-like growth factor; T3, triiodothyronine; and GAS, gastrin

To investigate the effects of ATC supplementation on the factors associated with nutrient intake in the pigs, we evaluated the protein levels of serum insulin (INS), insulin-like growth factor (IGF), triiodothyronine (T3), and gastrin (GAS). ATC supplementation significantly increased the levels of INS (*P* = 0.0018), IGF (*P* = 0.0018), and T3 (*P* = 0.0031) but did not affect the level of GAS (Fig. 1).

### Antioxidant status

**Fig. 2:**
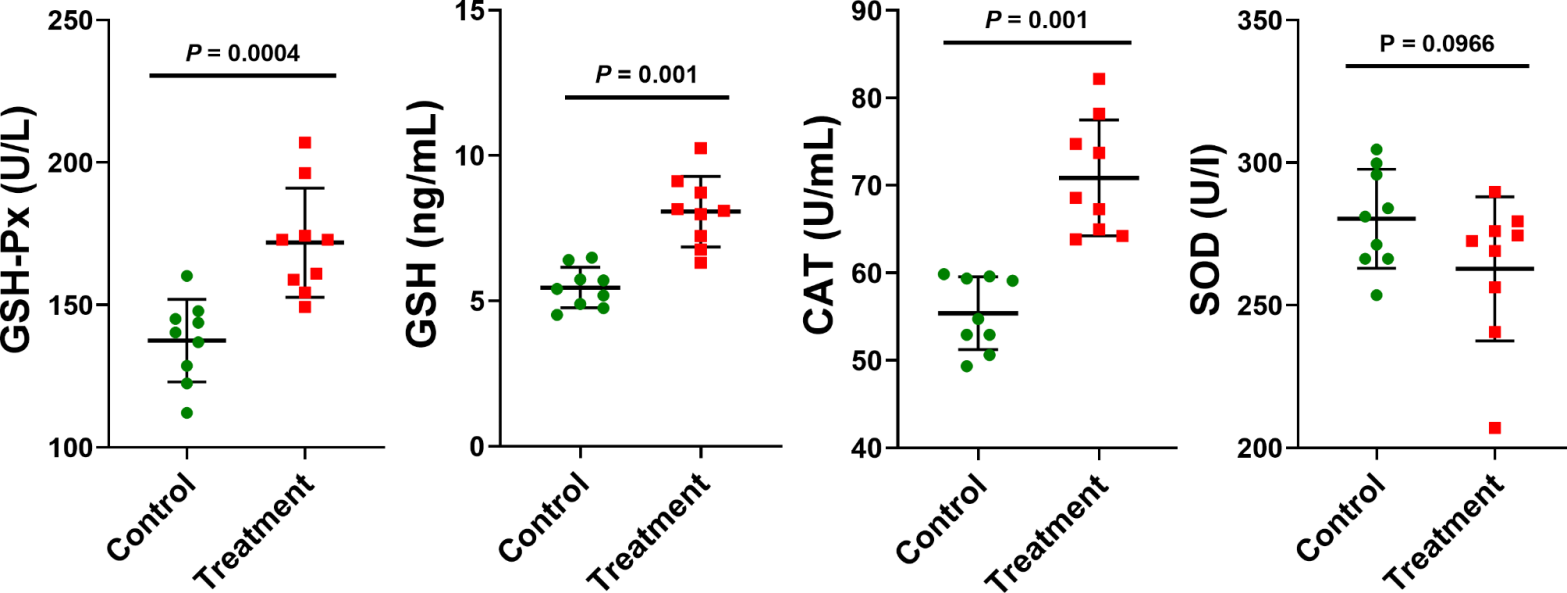
Effects of dietary supplementation with ***Acremonium terricola*culture on the antioxidant status of weaning piglets.** GSH: glutathione; GSH-Px, glutathione peroxidase; CAT, catalase; SOD, superoxide dismutase; and T-AOC, antioxidant capacity

Compared with the control group, the experimental group exhibited higher levels (activity) of glutathione (GSH), GSH peroxidase (GSH-Px), and catalase (CAT) but a lower level of superoxide dismutase (SOD) (*P* < 0.0001; Fig. 2). No significant difference was noted between the groups in total antioxidant capacity (T-AOC; *P* = 0.7965).

### Immunity

**Fig. 3:**
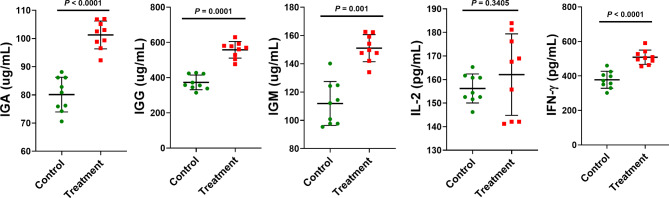
Effects of dietary supplementation with ***Acremonium terricola*culture on the immune function of weaning piglets.** IgA, immunoglobulin A; IgG, immunoglobulin G; IgM, immunoglobulin M; IFN-γ, Interferon-γ; and IL-2, interleukin-2

Higher immunoglobulin (Ig)A, IgG, IgM, and interferon (IFN)-γ levels were observed in the experimental group than in the control group (*P* < 0.001; Fig. 3). No significant difference was noted between the groups in the level of the inflammatory cytokine IL-2 (*P* = 0.3405). Thus, dietary supplementation with ATC modulated the immune status of weaning piglets by increasing the serum levels of major immune factors.

### Fecal microflora

**Fig. 4:**
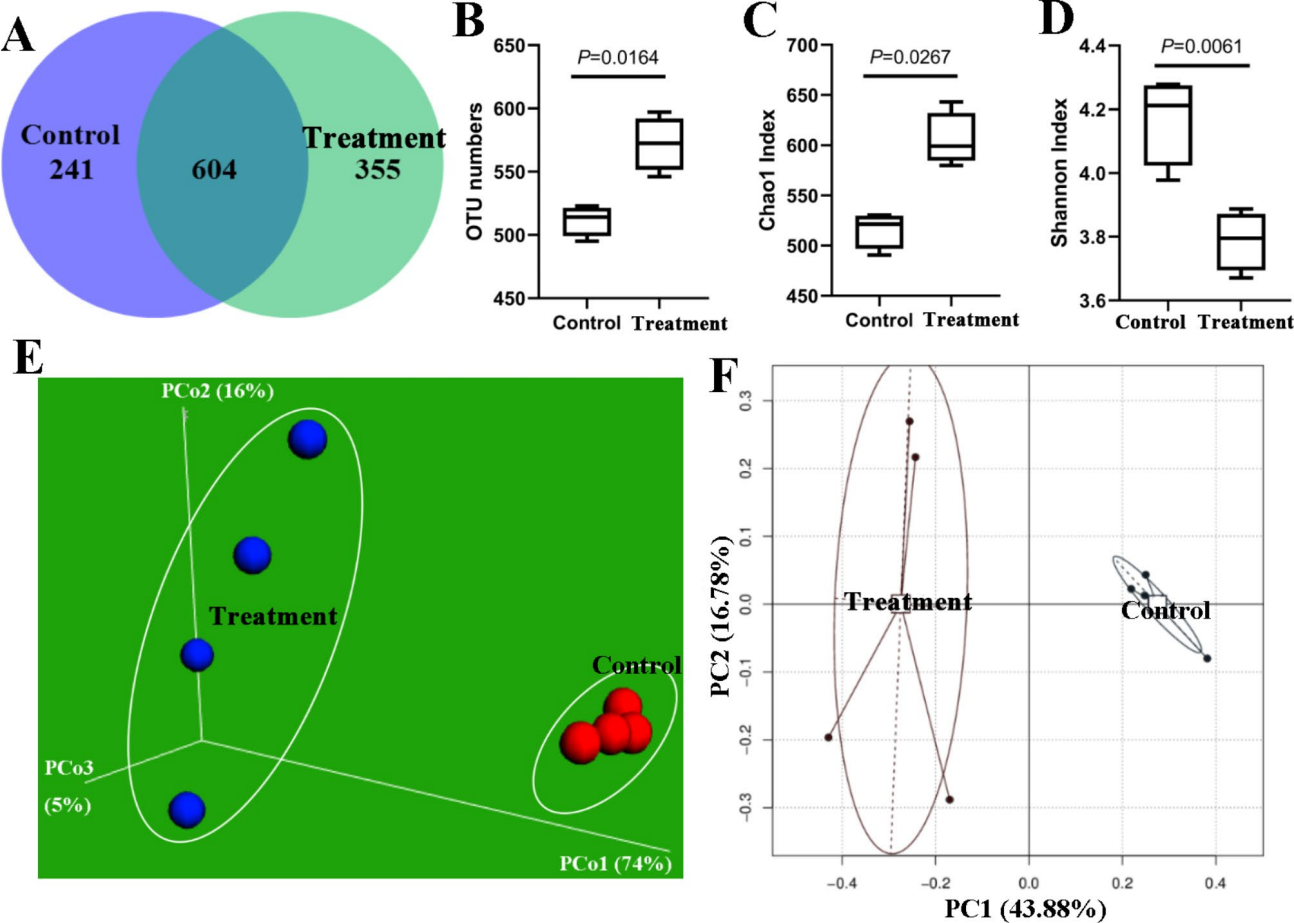
Effects of dietary supplementation with ***Acremonium terricola*culture on the gut microbial structure of weaning piglets.** (**A**) Venn diagram. (**B**) Operational taxonomic units. (**C**) Chao1 index. (**D**) Shannon index. (**E**) Plots constructed using data obtained through weighted UniFrac-based principal coordinate analyses. (**F**) Plots constructed using data obtained through principal coordinate analyses

We constructed a Venn diagram to explore the similarities and differences between the two groups in terms of microbial communities. The fecal microbial communities of the two groups had 604 common operational taxonomic units (OTUs); the experimental and control groups had 355 and 241 group-specific OTUs, respectively (Fig. 4A). ATC supplementation significantly (*P* < 0.05) increased the number of OTUs (Fig. 4B). Furthermore, α-diversity measured using the Chao1 index was significantly higher in the experimental group than in the control group (*P* < 0.05); however, contrasting results were obtained for α-diversity measured using the Shannon index (Fig. 4C,D). The two groups had significantly different microbiota profiles (*P* < 0.05; Fig. 4E,F).

### Bacterial classification

**Fig. 5:**
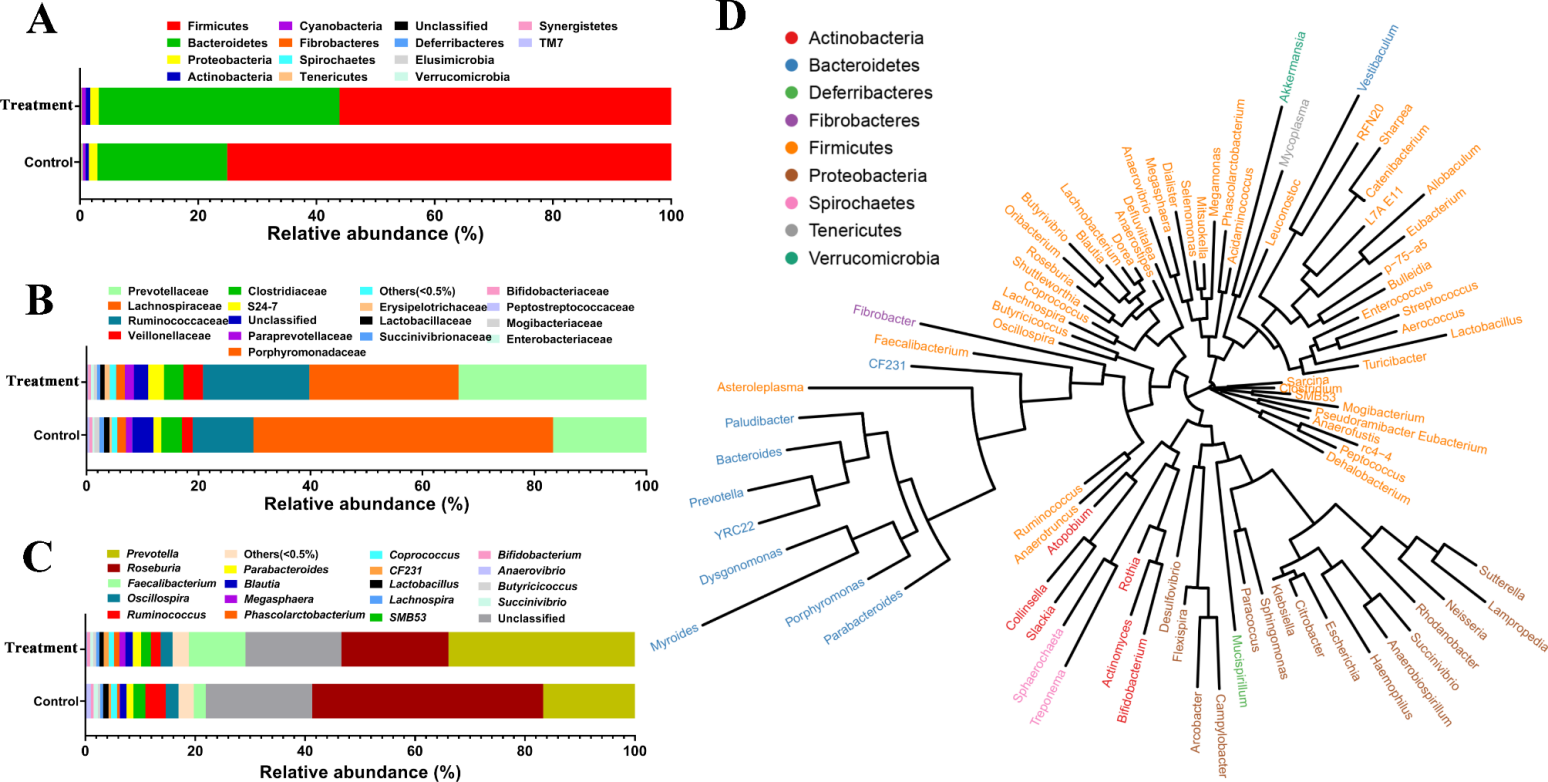
Effects of dietary supplementation with ***Acremonium terricola*culture on the gut microbiota composition of weaning piglets.** Bacterial profiles at the (**A**) phylum, (**B**) family, and (**C**) genus levels. (**D**) Results of the phylogenetic analysis (genus level) of the bacteria noted in the fecal samples of weaning piglets

Figure 5 presents the relative abundance of gut bacteria (at phylum, family, and genus levels) in the experimental and control groups. At the phylum level, the gut microbiota of the experimental group primarily comprised Firmicutes (56.1%), Bacteroidetes (40.7%), Proteobacteria (1.5%), and 11 other phyla (collectively comprised 1.7% of the total sequences analyzed). Significant increases and decreases were noted in the relative abundance of Firmicutes (75.1%) and Bacteroidetes (21.9%), respectively, in the control group compared with the findings in the experimental group (*P* < 0.05; Fig. 5A). At the family level, the five most abundant bacterial families (Fig. 5B) in the experimental group were Prevotellaceae (33.6%), Lactobacillaceae (26.6%), Ruminococcaceae (19%), Veillonellaceae (3.5%), and Clostridiaceae (3.5%). In the control group, the relative abundance of Lactobacillaceae significantly increased (*P* < 0.05) by 26.9%, whereas that of Prevotellaceae significantly decreased (*P* < 0.05) by 16.8%. At the genus level, *Roseburia* and *Prevotella* were the two most significantly (*P* < 0.05) enriched genera in the control group, whereas *Prevotella* was the most abundant genera in the experimental group (Fig. 5C).

Phylogenetic analysis at the phylum level revealed relationships between different microbial communities (Fig. 5D). Most categories of microbial communities belonged to the phylum Firmicutes and formed one cluster.

### Fecal microbiota dynamics

**Fig. 6:**
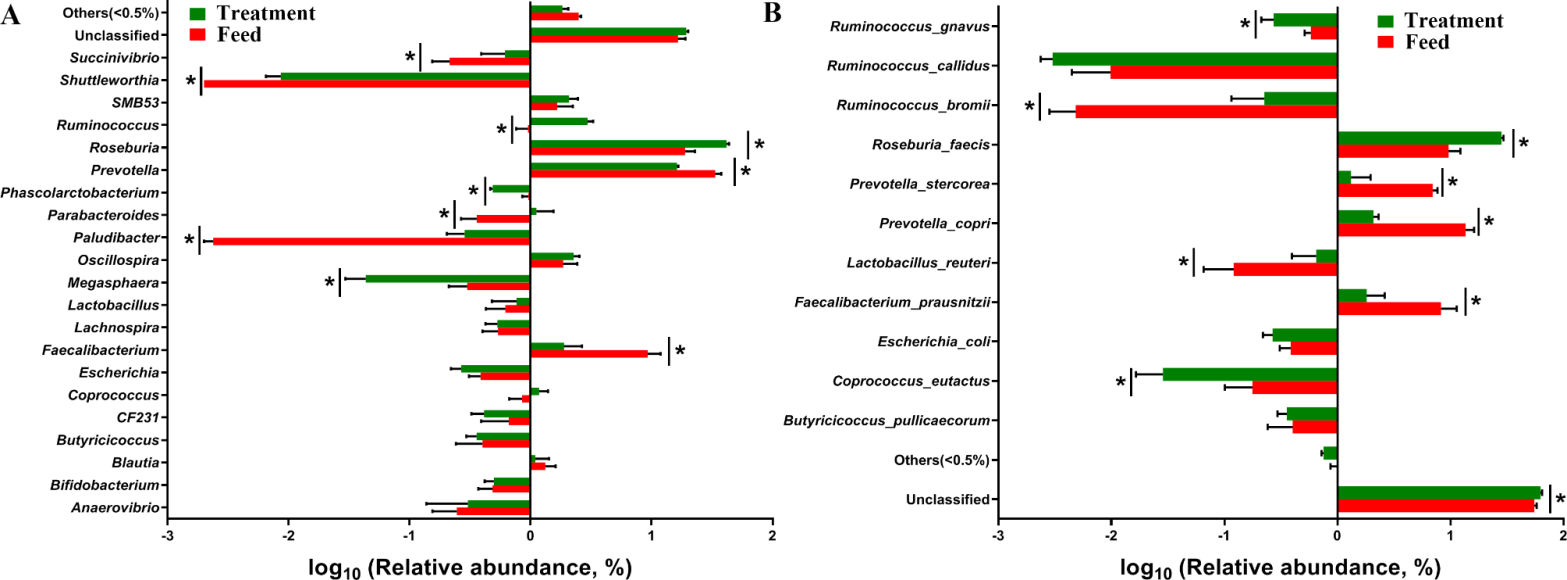
Effects of the dietary supplementation with *Acremonium terricola* culture on weaning piglets’ gut microbiota dynamics at the (**A**) genus and (**B**) species levels

Figure 6 illustrates the specific changes in the microbial composition of the two groups. At the genus level, ATC supplementation significantly (*P* < 0.05) reduced the relative abundance of *Shuttleworthia*, *Succinivibrio*, *Roseburia*, *Ruminococcus*, and *Paludibacter* but increased that of *Phascolarctobacterium*, *Megasphaera*, *Faecalibacterium*, and *Prevotella* in the experimental group (Fig. 6A). At the species level, ATC supplementation significantly increased the relative abundance of *Ruminococcus gnavus*, *Coprococcus eutactus*, *Prevotella copri*, *P. stercorea*, and *F. prausnitzii* but reduced that of *R. bromii* and *Roseburia faecis* (*P* < 0.05; Fig. 6B).

## Discussion

### Effects of ATC on growth performance

ATC has been used as an effective feed additive for ducks, piglets, and calves [14–19]. ATC supplementation accelerates weight gain in piglets, geese, rats, and weaned calves [14–19]. In the present study, no significant effect of ATC supplementation was observed on the amount of feed intake; ATC supplementation improved body weight by increasing feed efficiency. This finding is consistent with those of a previous study [19]. This observation indicates that increasing feed efficiency is crucial for improving body weight. A study indicated that higher levels of antioxidants and higher immunity can be attributed to ATC-mediated increases in the body weight and feed efficiency of calves [19], which is consistent with the findings of our study. INS, IGF, and T3 are essential for nutrition absorption and metabolism. ATC supplementation significantly increased the levels of INS, IGF, and T3, suggesting that ATC promotes the secretion of these proteins in piglets; this finding is in line with those of another study [20]. ATC contains various bioactive compounds, such as cordycepic acid, cordycepic polysaccharide, and ergosterol, which are involved in increasing the growth performance and feed efficiency of piglets [21].

### Effects of ATC on antioxidant status and immune function

Animals often develop oxidative stress–related diseases. In the present study, ATC supplementation increased the antioxidant status and immune function of piglets; this finding is supported by those of study conducted in weaned calves [19]. The cordycepic polysaccharide and other immunopolysaccharides, which are also the key bioactive compounds of ATC, may exert antioxidant effects and reduce lipid peroxidation [22, 23]. Although the functions of the indicated bioactive compounds of ATC have been clarified previously [19], the mechanisms underlying the antioxidant effects of ATC remain unclear. The cordycepic polysaccharide may enhance the activity of GSH-Px, contributing to the antioxidant property of ATC [21]. Reducing the expression levels of proinflammatory cytokines may protect organisms from excessive inflammation [19]. In our study, ATC supplementation increased the levels of IgA, IgG, IgM, and IFN-γ in the piglets, which indicates the ATC supplementation–mediated enhancement of immune function; this finding is similar to that of a study conducted in calves [24, 25].

### Effects of ATC on fecal microflora dynamics

Gut microbiota plays important roles in the promotion of animal health and growth, and the diet strongly influences the composition of gut microbiota [25, 26]. In the present study, Firmicutes and Bacteroidetes were the two predominant phyla in both the experimental and control groups; this observation is consistent with those of previous studies [27, 28]. ATC increased the relative abundance of Firmicutes but reduced that of Bacteroidetes, thus increasing the ratio of Firmicutes to Bacteroidetes. This finding indicated ATC supplementation mediated increases in body weight through enhanced lipid accumulation [29]. Notably, ATC supplementation substantially increased the relative abundance of *F. prausnitzii*, which is regarded as a “sentinel of the gut” because of its involvement in anti-inflammatory activities, gut barrier enhancement, and butyrate production [30]. This result further confirms that ATC alters the gut microbial structure of weaning piglets.

## Conclusions

Dietary supplementation with ATC may improve the ADG, feed efficiency, antioxidant status, and immunity of weaning piglets. ATC supplementation may further contribute to the intestinal health of these piglets by altering their gut microbiota, for example by increasing the relative abundance of *F. prausnitzii*. Thus, ATC may be an effective feed additive for weaning piglets. Our findings can improve pig farming practices and boost the associated economic gain.

## Methods

All animals experiments were conducted in accordance with the Animal Care and Use Committee guidelines of Anhui Agricultural University (approval number: AAU 2018 − 157; date: Jan 25, 2018) and the ARRIVE guidelines (https://arriveguidelines.org/).

### Animals and experimental design

Piglets were housed in pens with slatted concrete flooring (stocking density, 0.9 m^2^/pig). The pens were equipped with a low-pressure nipple drinker, stainless steel trough, and heating lamp. The feed composition and nutrient levels were set according to the recommendations of the National Research Council (2012). All piglets were vaccinated against Aujeszky’s disease, salmonellosis, and transmissible gastroenteritis.

A total of 120 piglets [(Landrace × Yorkshire) × Duroc; body weight, 11.8 ± 0.04 kg; mixed sexes] were weaned at 42 days of age (11.8 kg BW; SEM: 0.03 kg). The piglets were randomly and blinded assigned to two groups (experimental and control); three replicate pens (20 piglets per pen) were used per group (day 0 of the trial - December 2020). Piglets were assigned individually (within litters) to the treatment groups. At admission, the animals were ear tagged as they came to hand and were allocated with the use of a computer-generated randomisation list (only known to BH) to one of the two treatment groups, until the required number of piglets had been reached. Animal feeding was conducted in a commercial pig farm (Jiangxi Zhengbang Technology Co., Ltd., Jiangxi, China). During the 20-day-long experimental period, the control group was fed a basal diet, whereas the experimental group was fed the same basal diet supplemented with 1 g ATC/kg of basal diet according to a previously described method [31]. Table 2 lists the nutrient and chemical compositions of the basal diet. All piglets had free access to feed and water throughout the experimental period. Body weight and feed intake were measured every week. Pen-based feed disappearance was recorded to evaluate ADG, ADFI, and FCR. Only clinically healthy piglets were included in the study. Diseased piglets, runts and animals with abnormalities (e.g. hernia) were excluded. In total, all pigs from both the treatment and control groups reached the end of the study.

**Table 2:** Nutrition formulation of basic diet

Ingredient	Content (%)	Chemical composition	Content
corn	63.58	Digestive energy (MJ / kg)	13.5
Bean flour	19.1	Crude protein (%)	18.66
Extruded soybean, CP 35.5%	8	Crude fiber (%)	2.07
Fishmeal, CP 59.2%	2	Calcium (%)	0.62
Soybean oil	1.82	Phosphorus (%)	0.5
Premix ^a,b^	5.5	Salt (%)	0.3
Total	100	Lysine ^c^ (%)	0.13

^a^ Supplied, per kilogram of diet: vitamin A, 13,000 IU; vitamin B1, 3.0 mg; vitamin B2, 5 IU; vitamin B12, 0.025 mg; vitamin D2, 3000 IU; vitamin D3, 2500 IU; vitamin E, 30 IU; vitamin K3, 3 mg; Fe, 100 mg; Cu, 20 mg; Zn, 100 mg; Mn, 50 mg from manganese oxide; Se, 0.30 mg

^b^ Vitamins and minerals from Volac International, Liverpool, UK

^c^ Lysine from Qingdao Agri-King Industrial Co., Ltd., Shandong, China

### Blood collection and analyses

On Day 20, blood samples were collected from the piglets after they had fasted for 12 h. For blood collection, 10 piglets were randomly selected from each group. The samples were collected in 10-mL evacuated blood collection tubes containing heparin sodium and then centrifuged at 2000 ×*g* for 10 min. The samples were prepared following a previously described method [32]. The plasma obtained through the aforementioned centrifugation was frozen until use. The plasma levels of GSH, GSH-Px, SOD, CAT, and T-AOC were measured using commercial colorimetric analysis kits (Nanjing Jiancheng Institute of Bioengineering, Nanjing). In addition, the levels of porcine IgA, IgG, IgM, IL-2, and IFN-γ were measured through enzyme-linked immunosorbent assay (ELISA; Sangon Biotech Co., Ltd., Shanghai). IgA, IgG, and IgM were evaluated through a double-antibody sandwich ELISA specific for porcine Igs; the obtained data were compared with standard curves generated using purified swine IgA, IgM, and IgG (Bethyl Laboratories) antibodies, as described previously [33]. The protein levels of INS, IGF, T3, and GAS were measured using commercial ELISA kits (Shanghai Langdun Biotechnology Co., Ltd., China), as described previously [34]. All assays were performed in triplicate and according to the corresponding manufacturers’ instructions.

### Gut microbiota analysis

At the end of the intervention, fecal samples were collected from four piglets selected randomly from each group. The samples were collected through rectal dilation in plastic containers (50-mL conical centrifuge tubes). Genomic DNA was isolated from the eight fecal samples by using a DNA extraction kit (Omega, Inc., USA) according to the manufacturer’s instructions. The samples were treated with RNase to degrade RNA, and the quality and quantity (concentration) of DNA were evaluated through agarose gel electrophoresis and NanoDrop spectrophotometry. Sequencing libraries were established for the 16 S rRNA gene (V3 to V4 region) according to the relevant Illumina protocol. Paired-end shotgun sequencing was performed on the Illumina Hi-Seq 2000 platform. After the elimination of low-quality sequences, the remaining sequences were clustered into OTUs on the basis of ≥ 97% similarity assessed using Vsearch (version 2.3.4). Representative sequences were selected for each OTU, and taxonomic data were assigned to each representative sequence by using the Ribosomal Database Project Classifier, as described in a relevant study [35]. OTU abundance data were normalized using the standard sequence number corresponding to the sample with the least number of sequences. Alpha and beta diversity values were calculated through principal coordinate and cluster analyses performed using QIIME (version 1.8.0) [36].

### Statistical analysis

The pig was the statistical unit and the level of significance was set at 0.05. Sample size calculations were based on expected prevalence parameters, an expected coefficient of correlation of 0.5, α-value of 0.05, and power of 0.80. Data were analyzed using generalized linear models (SAS; version 9.4; SAS Institute, Cary, NC, USA). A randomized complete block design was used with “pen” as the experimental unit for the evaluation of growth performance. Each pig was used as an experimental unit for the analysis of blood parameters and microbial counts. The significance of between-group differences was determined using Duncan’s multiple range test (SAS). All data are presented as mean ± standard error values. *P* < 0.05 indicated statistical significance.

## Data Availability

Most data generated or analyzed during the current study are included in this published article. The other data are available from the corresponding author on reasonable request.

## Abbreviations

ATC: *Acremonium terricola* culture
BW: Body weight
CAT: Catalase
GSH-Px: Glutathione peroxidase, SOD:superoxide dismutase
GSH: Glutathione
IFN-γ: Interferon γ
IgA: Immunoglobulin
IgG: Immunoglobulin G
IgM: Immunoglobulin M
IL-2: Interleukin-2

## References

[1] Zhao Y (2020). Effects of GABA supplementation on intestinal SIgA secretion and gut microbiota in the healthy and ETEC-infected weanling piglets. Mediat Inflamm.

[2] Lee HJ (2021). Impact of supplementary microbial additives producing antimicrobial substances and digestive enzymes on growth performance, blood metabolites, and fecal microflora of weaning pigs. Animals.

[3] Li H (2021). Evolution of the gut microbiota and its fermentation characteristics of ningxiang pigs at the young stage. Animals.

[4] Tremaroli V, Bäckhed F (2012). Functional interactions between the gut microbiota and host metabolism. Nature.

[5] De Lange C (2010). Strategic use of feed ingredients and feed additives to stimulate gut health and development in young pigs. Livest Sci.

[6] Lalles JP, Montoya CA (2021). Dietary alternatives to in-feed antibiotics, gut barrier function and inflammation in piglets post-weaning: where are we now?. Anim Feed Sci Tech.

[7] Qiao J (2015). Effects of *Lactobacillus acidophilus* dietary supplementation on the performance, intestinal barrier function, rectal microflora and serum immune function in weaned piglets challenged with *Escherichia coli* lipopolysaccharide. Anton Leeuw Int J G.

[8] Finamore A (2014). *Lactobacillus amylovorus* inhibits the tlr4 inflammatory signaling triggered by enterotoxigenic *Escherichia coli* via modulation of the negative regulators and involvement of tlr2 in intestinal Caco-2 cells and pig explants. PLoS ONE.

[9] Wang Y (2019). *Lactobacillus casei* Zhang prevents jejunal epithelial damage to early-weaned piglets induced by *Escherichia coli* K88 via regulation of intestinal mucosal integrity, tight junction proteins and immune factor expression. J Microbiol Biotechnol.

[10] Che L (2017). Effects of dietary live yeast supplementation on growth performance, diarrhoea severity, intestinal permeability and immunological parameters of weaned piglets challenged with enterotoxigenic *Escherichia coli* K88. Brit J Nutr.

[11] Cheng YH (2016). Fermentation products of *Cordyceps militaris* enhance performance and modulate immune response of weaned piglets. S Afr J Anim Sci.

[12] Boontiam W (2020). Effect of spent mushroom (*Cordyceps militaris*) on growth performance, immunity, and intestinal microflora in weaning pigs. Animals.

[13] Giraldo A (2015). Phylogeny of *Sarocladium* (Hypocreales). Persoonia.

[14] Li Y (2017). Effects of *Acremonium terricola* culture supplementation on apparent digestibility, rumen fermentation, and blood parameters in dairy cows. Anim Feed Sci Tech.

[15] Lia Y (2018). Effects of *Acremonium terricola* culture on performance, milk composition, rumen fermentation and immune functions in dairy cows. Anim Feed Sci Tech.

[16] Chen JY (2022). Effects of *Acremonium terricola* culture on the growth, slaughter yield, immune organ, serum biochemical indexes, and antioxidant indexes of geese. Animals.

[17] Li Y (2016). Effects of Acremonium terricola culture on growth performance, antioxidant and immune indexes of serum and liver in Sprague Dawley rats (in Chinese). Chin J Anim Nutr.

[18] Wei J (2009). Effects of the culture of *Acrermonium Terricola* on performance and immunity of piglets (in Chinese). China Anim Husb Veterinary Med.

[19] Li Y (2016). Effects of *Acremonium terricola* culture on growth performance, antioxidant status and immune functions in weaned calves. Livest Sci.

[20] Dänicke S (2000). Farm animal metabolism and nutrition. Anim Feed Sci Tech.

[21] Zhang YM, Zhang Q, Liang ZQ (2006). Research situation and development trends of *Cordyceps gunnii* (in Chinese). Guizhou Agricultural Sciences.

[22] Jin MC, Huang WQ (2019). Research progress of *Cordyceps* feed additives in animal feed. Feed Res (in Chinese).

[23] Lee JS (2010). Structural characterization of immunostimulating polysaccharide from cultured mycelia of *Cordyceps militaris*. Carbohyd Polym.

[24] Moore E (2002). Expression of IL-17B in neurons and evaluation of its possible role in the chromosome 5q-linked form of Charcot-Marie-Tooth disease. Neuromuscul Disorders Nmd.

[25] Crespo-Piazuelo D (2019). Association between the pig genome and its gut microbiota composition. Sci Rep-UK.

[26] Ijaz MU (2018). Beef, casein, and soy proteins differentially affect lipid metabolism, triglycerides accumulation and gut microbiota of high-fat diet-fed C57BL/6J mice. Front Microbiol.

[27] Hou L (2021). Effects of protein restriction and subsequent realimentation on body composition, gut microbiota and metabolite profiles in weaned piglets. Animals.

[28] Wang X (2019). Longitudinal investigation of the swine gut microbiome from birth to market reveals stage and growth performance associated bacteria. Microbiome.

[29] Wang XX (2019). Amelioration of growth performance, lipid accumulation and intestinal health in mice by a cooked mixture of lean meat and resistant starch. Mol Nutr Food Res.

[30] He X, Zhao S, Li Y (2021). *Faecalibacterium prausnitzii*: a Next-Generation Probiotic in Gut Disease Improvement. The canadian journal of infectious diseases & medical microbiology = Journal canadien des maladies infectieuses et de la microbiologie medicale /. AMMI Can.

[31] Feng LL (2019). Effects of Acremonium terricola culture on growth performance, intestinal flora and humoral immunity in weaned piglets (in Chinese). Anim Husb Veterinary Med.

[32] Boontiam W, Wachirapakorn C, Wattanachai S (2019). Growth performance and hematological changes in growing pigs treated with *Cordyceps militaris* spent mushroom substrate. Veterinary World.

[33] Collier CT (2011). Insulin-like growth factor-1 attenuates glucocorticoid suppression of pig lymphocyte function. Food Agr Immunol.

[34] Phaengphairee P (2023). Dietary supplementation with full–fat *Hermetia illucens* larvae and multi–probiotics, as a substitute for antibiotics, improves the growth performance, gut health, and antioxidative capacity of weaned pigs. BMC Vet Res.

[35] Chen L (2020). Polysaccharides isolated from *Cordyceps Sinensis* contribute to the progression of NASH by modifying the gut microbiota in mice fed a high-fat diet. PLoS ONE.

[36] Caporaso JG (2010). QIIME allows analysis of high-throughput community sequencing data. Nat Methods.

